# The Impact of Eggshell Colour on the Quality of Table and Hatching Eggs Derived from Japanese Quail

**DOI:** 10.3390/ani10020264

**Published:** 2020-02-07

**Authors:** Kamil Drabik, Justyna Batkowska, Kostiantyn Vasiukov, Adrian Pluta

**Affiliations:** Institute of Biological Basis of Animal Production, University of Life Sciences in Lublin 13 Akademicka St., 20–950 Lublin, Poland; kamil.drabik2@gmail.com (K.D.); vasiukovk@gmail.com (K.V.); adrian_pluta@yahoo.com (A.P.)

**Keywords:** egg quality, hatchability, spotted shell, blue shells, Japanese quail

## Abstract

**Simple Summary:**

The eggshell is the first element in the assessment of both table and hatching eggs. It may be influenced by many factors, i.e., the birds’ genotype, their utility type, rearing system, environmental conditions and feed mineral additives. However, the eggshell colour may affect the shell itself as well as both the quality of eggs and their biological value. Among the standard coloured eggs of Japanese quail, the eggs with a uniform shell can be found, in white to celadon colour. Consumers have no preferences in this regard, they are satisfied with the small size and taste of the egg. However, breeders believe that these eggs may be worse in the case of internal quality, both in terms of consumption and hatching. The aim of the study was to evaluate table and hatching eggs of Japanese quails (*Coturnix coturnix japonica*) depending on the eggshell colour. It seems that Japanese quail eggs with uniform “blue” shells do not appear to be of poorer quality than those with brown-spotted shells if they are intended for consumption. However, in the aspect of hatching eggs, the eggshell colour may modify the hatching results and body weight of the chicks obtained.

**Abstract:**

The aim of the study was to evaluate table and hatching eggs of Japanese quails (*Coturnix coturnix japonica*) depending on the eggshell colour. The research was carried out in two stages, in terms of table eggs’ quality and their biological value as hatching eggs depending on the eggshell colour. In both stages, 300 Japanese quail eggs were used in each (600 in total) divided into two equal groups: with a brown-spotted shell, with a uniform shell in shades of blue. In the 1st stage, quality characteristics of the whole egg (weight, specific gravity, proportions of particular elements), yolk (weight, colour, index), albumen (weight, height) and shell (colour, strength, weight, thickness, density) were evaluated. In the 2nd stage, eggs were incubated under standard conditions and following biological characteristics were analyzed: eggs fertility, embryo mortality, hatchability of fertile and set eggs, body weight of hatchlings and their proportion in egg weight. The shell colour, “blue” or spotted, of Japanese quail eggs, does not appear to influence their quality if they are intended for consumption. However, the hatching results and body weight of obtained chicks of Japanese quail may be affected by the eggshell colour.

## 1. Introduction

The eggshell provides protection against mechanical damage and microbiological infection, it also regulates the water and gas exchange between the embryo and the external environment, as well as constituting the calcium source for the developing embryo) [1]. The quality of eggshells may be influenced by many factors, but the most important are: the genotype of the birds, their utility type, rearing system, environmental conditions [2] and mineral additives to the feed.

The eggshell is also the first element in the assessment of both table and hatching eggs. As a kind of table eggs “packaging”, it is one of the first parameters of consumer evaluation and the choice criteria, wherein the preferred colour varies, depending on the area where the research was conducted [3]. The analysis of consumer preferences in the field of eggs concerns almost solely chicken eggs. In the case of quail eggs, the uniform eggshell and the lower availability of the commodity on the market limit purchasing choices.

Egg colour depends on three basic dyes, i.e., protoporphyrin, biliverdin and its zinc chelate, which in various combinations give all possible shades of shells [4]. In eggs with blue or green shells, biliverdin and biliverdin chelate with zinc have a greater proportion, whereas in eggs with a brown shade shells, protoporphyrin dominates [5]. Both biliverdin and protoporphyrin are synthesized in the shell gland of the oviduct and then deposited simultaneously on the eggshell, with the highest intensity of staining observed at the end of the eggshell formation [6]. Research also indicates variability in the area of shell dyes deposition depending on their type [7].

It was confirmed that the eggshell colour may affect both the quality of eggs and their biological value. In blue-shelled eggs obtained from pheasants, greater activity of lysozyme was found, in relation to eggs with other shades of shell [8]. Other works [3,9] also showed the relationship between shell colour and albumen quality.

Shell traits may also affect hatchability and embryo development in domestic birds. Due to the fact that the egg is a closed system in terms of the mineral presence, the shell constitutes a source of building material necessary for a proper embryo development [10]. The relationship has been demonstrated between the intensity of shell pigmentation, its thickness and the chicken’s hatchability, which indicates a possible positive correlation between shell pigmentation processes and its calcification [11]. This may be conducive to better hatchability, as it has been shown that hatching results from eggs with a thicker shell can be up to 9% better than from eggs with a thinner one [12].

A characteristic trait of Japanese quail eggs is the spotted pattern on the shell. Interestingly, the arrangement of these spots is an individual feature for each female, enabling the identification of particular birds [13]. Among the standard coloured eggs, the eggs with a uniform shell can be found, in white to celadon colour, without spots [14]. Generally, consumers have no preferences in this regard. They are fully satisfied with the small size and taste of the egg [15]. However, breeders believe that these eggs may be worse in terms of the internal quality, both as regards consumption and hatching.

The aim of the study was to evaluate Table and hatching eggs of Japanese quails (*Coturnix coturnix japonica*) depending on the eggshell colour.

## 2. Materials and Methods

In the 1st stage of the research, the material consisted of 300 Japanese quail eggs obtained from a stock kept at the Laura Kaufman Small Animal Teaching and Research Station belonging to the University of Life Sciences in Lublin. The eggs were divided into two equal groups: with brown-spotted shell and with a uniform shell in shades of blue. The eggs were collected on the same day and numbered individually. Eggs with visible damage were not intended for analysis.

The quails were housed in battery cages system with sex ratio (1♂:4♀). They were fed with a diet containing 2900 kcal of metabolizable energy/kg and 20% of crude protein with free access to feed and water. Cage equipment and birds’ density met the standards stipulated in the Regulation of the Ministry of Agriculture and Rural Development dated 28 June 2010. This is in case of minimal conditions for keeping livestock species, other than those for protection standards which are specified in the regulations of the European Union. Environmental conditions complied with the guidelines which are organized according to the Regulation of the Ministry of Agriculture and Rural Development dated on 2 September 2003 in the case of minimal conditions for keeping particular species of livestock.

The electronic set EQM (Egg Quality Measurement, TSS^®^), Instron Mini 55 (American Instrument Exchange, Haverhill, MA, USA) apparatus and electronic caliper were used for the test. The characteristics of particular egg elements were evaluated: in the whole egg: weight (EW), index (as the ratio of long and short axis length), specific gravity (SG, based on dry and water egg weight measurement, according to Archimedes principle); in yolk: colour (YC, according to Roche’s scale, DSM^®^), weight (YW) and index (ratio of height to diameter); in albumen: height (AH), Haugh units (HU) [16], weight; in shell: strength (Instron 55 Mini), colour (S.C., expressed as a percentage of reflected light), weight (SW), thickness (ST) and density (SDD) calculated from the eggshell thickness and weight [17].

Three hundred eggs collected on the same day from the same stock as in the 1st stage were used for the 2nd stage of the study. The eggs were divided into two groups: with a brown-spotted shell and with a uniform shell in shades of blue colour. They were placed on two separate hatching trays (150 eggs each), separating replication subgroups of 10 eggs each. The eggs were described individually and then subjected to the standard disinfection procedure (formaldehyde fumigation). Then the eggs were placed in a Jarson automatic incubator (Jarson^®^, Gostyń, Poland) and incubated. The following conditions were kept:

- 37.6–38.0 °C temp. and 50%–65% relative humidity in the setting compartment, eggs were turned automatically by 90° every 3 h (8 times a day).
- 37.0–37.5 °C temp. and 75%–80% relative humidity in hatching compartment.

On the 14th day of incubation, the eggs were candled and the number of infertile eggs and or dead embryos were determined, as well as eggs being transferred to hatching nets and placed in the hatching compartment for 17.5 d. After the incubation, selected biological features of the set were analyzed, such as the number of fertile eggs, embryos that died in the early incubation phase, embryos the died in the second incubation phase, and the numbers of healthy, crippled or weak chicks. This allowed the estimation of the following indicators: fertility, hatchability from fertile and from set eggs.

Before the transfer between compartments the eggs were weighed, with the division into eggs with a living and dead embryo, infertile or unhatched. On this basis, water conductivity of the eggshell (mg H_2_O/d/mm Hg) was calculated [18]. The proportion of the chick body weight in the egg weight was also calculated.

The data obtained were statistically processed using the SPSS 24.0 statistical package [19]. The groups were compared using the F test and the Student *t*-test for independent samples. The relationships between the assessed traits of the tested eggs were also analyzed using Spearman’s correlation coefficients. Non-parametric test χ^2^ was used to analyze the dependence of the number of meat and bloody spots on the eggshell colour of the examined eggs. Hatching data, after verifying the normality of distribution (Kolmogorov–Smirnov test), were analyzed by the non-parametric Mann–Whitney test for independent samples (equivalent to Student’s *t*-test).

## 3. Results

Table 1 shows the variation in egg quality depending on the eggshell colour. It was observed that the spotted eggs had a significantly higher weight and percentage of yolk than the blue ones. In contrast, blue eggs had a considerably higher proportion of albumen. The egg index, its specific gravity and percentage of shell did not differ significantly between groups of eggs depending on their shell colour. Brown-spotted eggs were characterized by a significantly higher shell strength and larger surface area than blue eggs. The colour of the shell did not significantly differentiate the weight, density and volume of this morphological element. Analyzing the yolk parameters, it was found that the spotted eggs had smaller yolks and considerably lower its index, which means that they were less spherical than in blue-shelled eggs.

**Figure 1:**
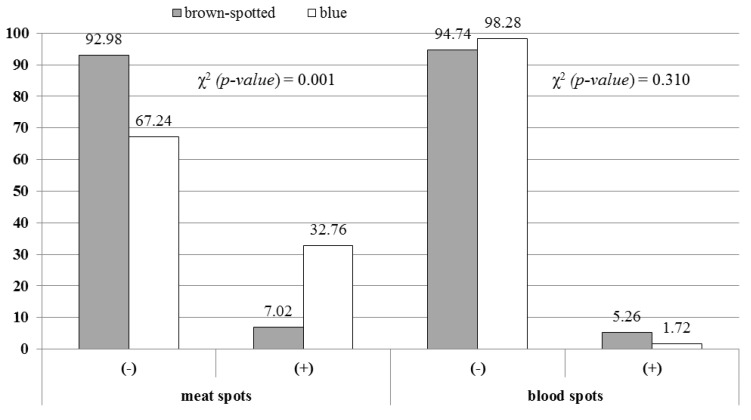
Proportions of Japanese quail eggs with meat and blood spots depending on the eggshell colour.

Figure 1 presents the percentage of quail eggs in which the presence of meat and blood spots was stated depending on the eggshell colour. It was shown that blue eggs had a significantly higher percentage of meat spots, while in spotted eggs a higher frequency of blood spots was observed.

On the basis of the data obtained, the relationships between the quality characteristics of table eggs from Japanese quail were determined (Table 2). A significant negative correlation between the refractive eggshell colour and the egg and yolk weight was found. For albumen height, a positive relationship was noted (0.423 at *p* ≤ 0.01). The egg weight and shell density were also negatively correlated. The specific gravity was positively correlated with the shell strength and thickness as well as the yolk weight and colour. Interestingly, both the shell weight as well as its density were features negatively correlated with the albumen weight (−0.365 and −0.416, respectively).

Table 3 summarizes the results of hatching quail eggs depending on their shell colour. A slightly higher proportion of fertile eggs was characteristic for eggs from spotted group. Also, in this group the hatchability from set eggs was significantly higher than from blue-shelled eggs. Both the proportion of fertile eggs, the number of dead embryos in the 1st and 2nd phase of hatching, or the hatchability of fertile eggs did not differ considerably between the tested groups. Additionally, the chicks classified as crippled hatched only from blue-shelled eggs.

The changes in the weight of incubated quail eggs depending on the eggshell colour are presented in Table 4. Significantly higher initial mass, as in the case of table eggs, were characteristic for eggs with brown-spotted shell. Analyzing the 1st phase of hatching, a considerably higher eggs weight with an alive embryo in blue eggs and their higher water conductivity were observed. Spotted eggs had a significantly higher percentage weight loss of eggs with a living embryo. In the 2nd incubation phase no statistical differences were observed between tested groups of eggs. From eggs with spotted shells chicks with a higher body weight were obtained than from eggs with uniform ones, however, the proportion of the chick body weight in the initial egg weight was similar in both groups.

Table 5 presents Spearman’s correlation coefficients between measurable features of hatching Japanese quail eggs subjected to this study. The coefficients were estimated without taking into account the values of traits obtained for eggs identified as infertile, dead or unhatched. A statistically significant negative relationship was found between the shell colour and the initial egg weight, hatchability from set and fertile eggs and all indicators of the weight change for eggs with developing embryos. As the percentage of reflected light (shell colour) increased, the percentage loss and water conductivity of the eggshell containing live embryos increased with the decrease in the body weight of the chicks obtained. The initial egg weight determined such features as egg hatchability (positive relationship 0.454 at *p* ≤ 0.01) or body weight of chicks (0.650). Importantly, the eggshells’ colour was negatively correlated with the chicks hatchability from set eggs, the egg weight and the body weight of the hatched chicks. On the other hand, positive relations were noted between the water conductivity of the shell and the water loss during incubation depending on the eggshell colour.

## 4. Discussion

In our research it was found that the eggshell colour considerably affects the weight of Japanese quail eggs. Data available in the literature are not uniform in this respect. Research of Hassan et al. [20] did not show significant differences in the weight of quail eggs depending on the eggshell colour, even though eggs with greater colour variability were compared. The results obtained by Taha [9] are different, and significant differences in egg weight depended on the shell colour were found, however, eggs in spotted shells had a higher weight in comparison with blue-shelled eggs, which is compatible with our results. The differences may be caused by many reasons, although the most obvious is the birds’ ages. Zita et al. [21] indicate a significant impact of the flock age on the weight of eggs laid by quails. In own research, eggs came from birds of the same age, which allowed to omit this factor during the study. Additionally, the occurrence of celadon mutation, visible as blue colour of the quail eggshell, is not linked with the age of the flock [14].

The shell colour or pigmentation of the quail egg (black, brown, blue, speckled and white) may determine its weight and shape index, as well as the yolk index, Haugh units, weight loss during storage and proportions of particular morphological elements [9]. The effect of shell colour on the quality of egg content was also confirmed [22]. Similar observations were noted in our work. The results presented by Taha [9] showed a higher proportion of yolk weight in eggs with a spotted shell, compared to eggs with blue ones and, therefore, inversely than the results from own research.

Some differences in values of quality traits in some extent could be explained by considerably different egg weight. In chicken eggs it is confirmed that larger eggs are characterized by bigger albumen proportion as well as height of dense fraction of albumen [23]. However, in the case of quail eggs these relationships are not so unequivocal [24].

The eggshell colour also affects the quality characteristics of the shell itself. Own research has shown that brown-shelled eggs were characterized by a more resistant shell with a bigger surface. This is interesting because the studies conducted previously have shown a relationship between the protoporphyrins amount and the shell strength. Gosler et al. [25] found such a kind of correlation in the great tit, and additionally in the work of García-Navas et al. [26] it was found that the dye accumulation in blue tit characterized places with a small amount of calcium. Analyzing the data, it was observed that the shell thickness did not differentiate eggs depending on its colour. It was similarly concluded by Sari et al. [22], however, Taha [9] reports that the thinnest shell is characteristic for spotted eggs, with the highest values for blue and black ones.

Some research suggests that thicker shell may result from more intensive calcification, darker eggs were characterized by bigger density and thickness of the shell [11], however these findings concerned chicken eggs. In guinea fowl among usually pigmented eggs, unpigmented shells are also observed. They are characterized by a smaller eggshell mass and its lower proportion in egg weight with no differences in porosity [27]. Knowledge about eggs derived from Japanese quails is rather limited.

In our research, on the basis of the data obtained, the relationships between selected external and internal characteristics and the whole egg were determined. It was found that the yolk colour is positively correlated with its weight. In the research of Yilmaz et al. [28] no such relationship was found. However, a correlation between the shell weight and the yolk weight was similar to our own research (0.253 vs. 0.366). In turn, according to the data presented by Alkan et al. [29], the shell weight and its thickness are significantly positively correlated. In our own research this tendency has not been confirmed.

The biological value of eggs is primarily understood as their traits related to the hatching efficiency i.e., hatchability, fertility rate and quality of chicks. Studies on other bird species have shown a relationship between the eggshell colour and the incubation results. Early studies of this phenomenon [30] conducted in chickens have shown that the hatching of chicks from eggs with very bright shells is much lower, compared to eggs with darker ones. Kożuszek et al. [31] came to similar conclusions. Their results on pheasant eggs examination showed that the results of hatching from brown and olive eggs are much better compared to blue-shade eggs. These observations are consistent with our own results, which showed lower hatchability from blue-shell eggs. Similar observations in case of quail eggs were made by Soliman et al. [32], who noticed that both hatching and fertility rates in the brightest eggs are lower than in eggs with darker shells. It should be noted that eggs with a uniform “blue” shell had significantly lower weight compared to eggs with a spotted one. Uddin et al. [33], analyzing the effect of egg weight on hatchability, found a positive relationship between both these parameters. It is possible, therefore, that the hatching results were influenced not only by the eggshell colour but also by the initial egg weight, which would explain the results of our own research.

Research conducted by McDaniel et al. [34] and Bennett [12] showed that pheasant eggs with blue or light brown shells showed considerably smaller hatchability. Moreover, thinner eggshells were associated with a reduction in the hatchability percentage, while eggs with a thick shell were characterized by better fertility, increased hatchability and lower embryo mortality [35,36]. Similar observations apply to our own research. Furthermore, studies by Baggott et al. [37] conducted on Houbara bustrad, showed that the colour of the shell affects its thickness and water conductivity. They found that darker shell are thicker and dark-shelled eggs show lower water loss during storage or incubation. A similar tendency was observed in our research in terms of both water conductivity and water losses from eggs during their incubation.

The main building material of the shell is calcium carbonate (96%), and the remaining components are magnesium, phosphorus, but also copper, zinc, iron [38] and many trace elements, among them lithium, strontium and bar [39]. All the described relationships, between the eggshell colour and hatchability, are probably due to properties of dyes, which give the eggshell the appropriate, characteristic colour and contribute to the mineral elements’ diversity of egg content and its shell. Probably, the mineral composition of the eggshell as well as the building elements’ bioavailability depend on its colour as well as affecting the biological value of hatching eggs [40]. However, this phenomenon needs further analysis including among various species of birds.

## 5. Conclusions

It was shown that Japanese quail eggs with a blue eggshell colour were significantly smaller than spotted eggs; they were also characterized by poorer shell strength, smaller and less spherical yolks while at the same time having better dense albumen height and Haugh units. However, no differences were found in the shell density or thickness. An interesting observation was made in the aspect of the meat spots’ presence in the eggs. In the group with brown-spotted shells, meat spots were found in 6.6% of eggs, while in the group with uniform blue-shade shells this was up to 24.6%. This relationship was confirmed statistically.

The share of fertile eggs and the number of dead embryos in the 1st and 2nd phase of incubation or hatchability from fertile eggs did not differ significantly between the groups of the examined eggs, regardless of their eggshell colour. However, it should be noted that crippled chicks hatched only from eggs with a blue colour of shell. Hatchability from set eggs was considerably higher in brown-spotted eggs than from blue shelled ones. Also, chicks hatched from spotted eggs were characterized by significantly higher body weight than those obtained from blue-shelled eggs.

It seems that Japanese quail eggs with uniform “blue” shells do not appear to be of poorer quality than those with a spotted shell if they are intended for consumption. However, in the aspect of hatching eggs, the colour of the eggshell may modify the hatching results and body weight of the chicks obtained.

## Figures and Tables

**Table 1:** The differentiation of quality characteristics of Japanese quail table eggs depending on the eggshell colour (mean ± standard deviation (SD)).

Trait	Eggshell	
	Brown-Spotted	Blue
Shell colour (%)	29.70 * ± 8.904	50.77 * ± 8.147
Whole egg		
Weight (g)	10.88 * ± 0.725	10.27 * ± 0.708
Index	1.308 ± 0.058	1.305 ± 0.052
Specific gravity (g/cm^3^)	1.06 ± 0.010	1.06 ± 0.006
Proportions (%)		
Shell	16.40 ± 4.176	16.14 ± 3.176
Albumen	48.42 * ± 8.906	52.29 * ± 6.893
Yolk	35.18 * ± 6.948	31.57 * ± 5.891
Shell		
Strength (N)	12.04 * ± 4.111	10.01 * ± 4.452
Weight (g)	1.81 ± 0.466	1.70 ± 0.365
Thickness (mm)	0.201 ± 0.049	0.197 ± 0.086
Surface area (cm^2^)	23.47 * ± 1.035	22.59 * ± 1.044
Volume (cm^3^)	0.472 ± 0.117	0.444 ± 0.185
Density (g/cm^3^)	4.01 ± 1.331	4.19 ± 1.403
Yolk		
Colour (pts)	7.44 ± 2.383	7.56 ± 1.191
Index (%)	38.01 * ± 4.196	41.52 * ± 4.659
Weight (g)	3.88 * ± 0.763	3.33 * ± 0.655
Albumen		
Height (mm)	9.47 * ± 1.473	9.40 * ± 1.622
HU	82.97 * ± 12.498	89.15 * ± 4.606
Weight (g)	5.38 ± 1.126	5.52 ± 0.816

*—differences are significant at *p* ≤ 0.05, HU—Haugh units.

**Table 2:** Relations (Spearman’s correlation coefficients) between measurable traits of Japanese quail table eggs.

Trait	SC	EW	SG	SS	SW	ST	SDD	YC	YW	AH
**EW**	−0.253 **									
**SG**	0.044	0.115								
**SS**	−0.027	0.210 *	0.502 **							
**SW**	−0.051	0.070	0.068	0.033						
**ST**	−0.169	0.217 *	0.226 *	0.418 **	−0.128					
**SDD**	0.079	−0.264 **	−0.071	−0.232 *	0.690 **	0.715 **				
**YC**	−0.120	0.036	0.409 **	0.076	0.292 **	−0.065	0.257 **			
**YW**	−0.316 **	0.510 **	0.207 *	0.156	0.253 **	0.086	0.053	0.246 **		
**AH**	0.423 **	0.035	−0.146	−0.080	−0.106	−0.126	−0.073	−0.208 *	−0.224 *	
**AW**	0.032	0.528 **	0.055	0.102	−0.365 **	0.106	−0.416 **	−0.173	−0.205 *	0.245 **

**—coefficient is significant at *p* ≤ 0.01 one-sided; *—coefficient is significant at *p* ≤ 0.05 one-sided; SC—shell colour; EW—egg weight; SG—specific gravity; SS—shell strength; SW—shell weight; ST—shell thickness; SDD—shell density; YC—yolk colour; YW—yolk weight; AH—albumen height; AW—albumen weight.

**Table 3:** The hatching results of Japanese quails’ eggs depending on their shell colour (mean ± SD).

Trait	Eggshell	
	Brown-Spotted	Blue
Shell colour (%)	30.11 * ± 7.399	52.71 * ± 5.740
Fertility (%)	96.67 ± 6.172	90.00 ± 12.536
Embryos died during the 1st phase of incubation (%)	7.33 ± 8.837	7.33 ± 7.037
Embryos died during the 2nd phase of incubation (%)	4.00 ± 5.071	6.00 ± 12.421
Crippled chicks (%)	0.00 * ± 0.000	6.67 * ± 8.165
Hatchability of fertile eggs (%)	86.43 ± 11.698	77.61 ± 14.019
Hatchability of set eggs (%)	84.00 * ± 11.832	68.67 * ± 12.459

*—differences are significant at *p* ≤ 0.05.

**Table 4:** Weight changes of incubated Japanese quail eggs depending on their shell colour (mean ± SD).

Trait		Eggshell	
		Brown-Spotted	Blue
**Initial Egg Weight (g)**		10.68 * ± 0.194	10.06 * ± 0.046
1st phase of incubation (up to 14th day)	Weight of infertile eggs (g)	7.69 ± 2.587	7.65 ± 1.601
	Weight loss of infertile eggs (%)	30.10 ± 23.553	30.40 ± 14.580
	Shell conductance of infertile eggs (mg H_2_O/d/mmHg)	4.89 ± 3.824	4.94 ± 2.367
	Weight of eggs with dead embryo (g)	8.29 ± 2.269	7.49 ± 1.105
	Weight loss of eggs with dead embryo (%)	26.62 ± 24.488	33.61 ± 10.097
	Shell conductance of eggs with dead embryo (mg H_2_O/d/mmHg)	6.45 ± 5.950	9.13 ± 5.652
	Weight of eggs with alive embryo (g)	9.34 * ± 0.245	8.70 * ± 0.499
	Weight loss of eggs with alive embryo (%)	15.00 * ± 2.229	20.89 * ± 4.537
	Shell conductance of eggs with alive embryo (mg H_2_O/d/mmHg)	2.44 * ± 0.362	3.40 * ± 0.737
2nd phase of incubation (up to 17.5th day)	Weight of unhatched eggs (g)	8.72 ± 0.811	8.73 ± 0.815
	Weight loss of unhatched eggs (%)	20.67 ± 7.392	20.65 ± 7.398
	Shell conductance of unhatched eggs (mg H_2_O/d/mmHg)	2.69 ± 0.958	2.68 ± 0.963
Chick weight (g)		6.47 * ± 0.394	6.05 * ± 0.169
Proportion of chick in initial egg weight (%)		60.08 ± 2.464	60.16 ± 1.644

*—differences are significant at *p* ≤ 0.05.

**Table 5:** Relations (Spearman correlation coefficients) between measurable traits of Japanese quail hatching eggs.

Trait	SC	EW	F	HFE	HSE	EWA	WLA	ESCA	BW
**EW**	−0.753 **								
**F**	−0.266	0.098							
**HFE**	−0.384 *	0.365 *	−0.075						
**HSE**	−0.514 **	0.454 **	0.429 **	0.799 **					
**EWA**	−0.589 **	0.753 **	0.080	0.433 **	0.463 **				
**WLA**	0.593 **	−0.757 **	−0.080	−0.431 **	−0.462 **				
**ESCA**	0.589 **	−0.753 **	−0.080	−0.433 **	−0.463 **				
**BW**	−0.457 **	0.650 **	−0.156	0.161	0.105	0.582 **	−0.587 **	−0.582 **	
**BW/EW**	0.192	0.005	−0.450 **	−0.167	−0.379 *	0.113	−0.115	−0.113	0.673 **

**—coefficient is significant at *p* ≤ 0.01 one-sided; *—coefficient is significant at *p* ≤ 0.05 one-sided; SC—shell colour; EW—egg weight; F—fertility; HFE—hatchability of fertile eggs; HSE—hatchability of set eggs; EWA—weight of eggs with alive embryo; WLA—weight loss of egg with alive embryo; ESCA—egg shell conductance of eggs with alive embryo; BW—body weight of healthy chick; BW/EW—proportion of chick in initial egg weight.
